# Clinical Translation of Long-Acting Drug Delivery Systems for Posterior Capsule Opacification Prophylaxis

**DOI:** 10.3390/pharmaceutics15041235

**Published:** 2023-04-13

**Authors:** Xinyang Li, Chen Liang, Yexuan Guo, Jing Su, Xi Chen, Robert B. Macgregor, Rui Xue Zhang, Hong Yan

**Affiliations:** 1 Xi’an People’s Hospital (Xi’an Fourth Hospital), Shaanxi Eye Hospital, Affiliated People’s Hospital of Northwest University, 21 Jiefang Road, Xi’an 710004, China; 2 Xi’an Key Laboratory of Stem Cell and Regenerative Medicine, Institute of Medical Research, Northwestern Polytechnical University, 127 West Youyi Road, Xi’an 710072, China; 3 Department of Pharmaceutical Sciences, Leslie Dan Faculty of Pharmacy, University of Toronto, 144 College Street, Toronto, ON M5S 3M2, Canada

**Keywords:** drug delivery, PCO, controlled release, pharmacological agent, dosage form, intraocular lens, implant, lens epithelial cell

## Abstract

Posterior capsule opacification (PCO) remains the most common cause of vision loss post cataract surgery. The clinical management of PCO formation is limited to either physical impedance of residual lens epithelial cells (LECs) by implantation of specially designed intraocular lenses (IOL) or laser ablation of the opaque posterior capsular tissues; however, these strategies cannot fully eradicate PCO and are associated with other ocular complications. In this review, we critically appraise recent advances in conventional and nanotechnology-based drug delivery approaches to PCO prophylaxis. We focus on long-acting dosage forms, including drug-eluting IOL, injectable hydrogels, nanoparticles and implants, highlighting analysis of their controlled drug-release properties (e.g., release duration, maximum drug release, drug-release half-life). The rational design of drug delivery systems by considering the intraocular environment, issues of initial burst release, drug loading content, delivery of drug combination and long-term ocular safety holds promise for the development of safe and effective pharmacological applications in anti-PCO therapies.

## 1. Introduction

A cataract is an eye disease in which the anterior segment of the lens becomes cloudy, causing visual impairment. Cataracts can occur in children and adults [1,2]. Cataract surgery is the only effective treatment and involves removing the crystalline lens fibers, followed by implantation of an artificial intraocular lens (IOL) in the capsular bag [3,4]. Despite lens removal after cataract surgery, within two to five years post-surgery, as high as 50% of the elderly and up to 100% of children develop posterior capsule opacification (PCO), known as a secondary cataract [5]. PCO ultimately encroaches on the visual axis, leading to blurry vision, poor visual acuity and even functional blindness [4,6]. Fundamentally, PCO is a post-surgical wound-healing response of the damaged capsule lens, in which residual lens epithelial cells (LECs) start to proliferate and migrate to denuded regions of both the anterior and posterior capsule as well as the IOL surface [4]. There are two forms of PCO, fibrotic and regenerative. Of these, fibrotic PCO is responsible for all crucial pathological processes of the visual axis, including the hyperproliferation and migration and epithelial–mesenchymal transition (EMT) of LECs and the matrix contraction and deposition. Compared to the fibrotic form, regenerative PCO is developed at a later stage following cataract surgery. The most severe forms of posterior and peripheral capsular opacification are Elschnig’s pearls and Soemmerring’s ring, respectively [4,6]. Clearly, PCO is an important problem in ophthalmological health.

Options for the clinical management of PCO currently include (1) implantation of specially designed IOLs (e.g., square-edged shape, hydrophobic AcrySof materials) to slow the progression of PCO, and (2) laser capsulotomy, which involves using laser light to make an opening in the opaque posterior capsule [7,8]. However, laser capsulotomy is expensive, and it is accompanied by complications such as intraocular inflammation, damage and altered position of the IOL and even the development of macular edema [9,10]. Pre-clinical and clinical studies have shown the efficacy of pharmacological agents, particularly chemotherapeutic drugs, against the proliferation and fibrosis of LECs [11,12,13]. Often, these drugs are directly administered for a short period into the capsular bag at the time of lens extraction followed by aspiration [11]. Although this approach is effective, concerns about collateral toxicity to normal ocular tissues (e.g., cornea) hinders pharmacological application [13,14].

**Figure 1:**
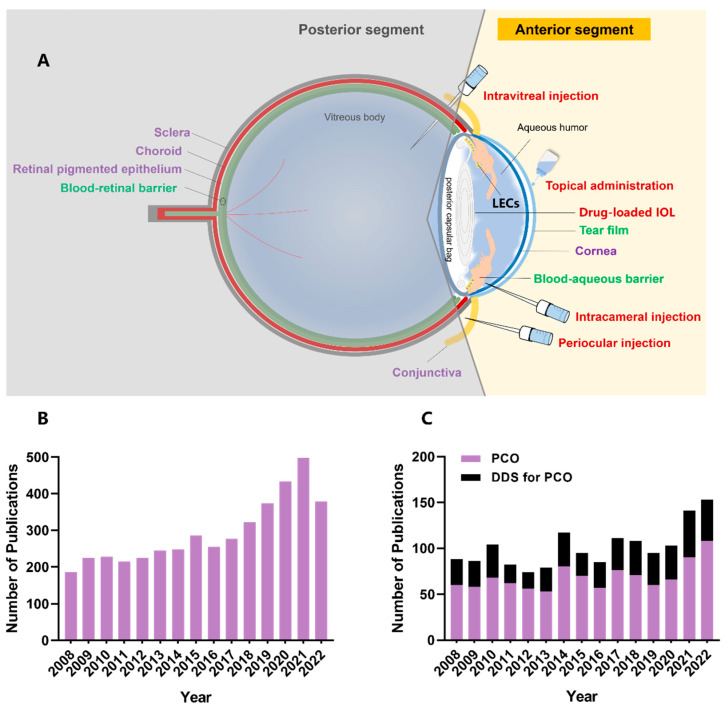
DDSs used for the anterior segment of the eye. (**A**) Ocular barriers to pharmacological agents and common routes of administration. Intricate ocular barriers physically and physiologically reduce intraocular drug bioavailability. The static barriers (purple colored text, including the cornea, conjunctiva, sclera, choroid and retinal-pigmented epithelium) prevent the drug from intraocular penetration, and the dynamic barriers (green colored text, including tear film, blood–aqueous barrier and blood–retinal barrier) lead to drug clearance. The drug can be locally administered to the anterior segment via eyedrops, injections (e.g., periocular, intracameral or intravitreal) and implants (e.g., drug-eluting IOL). (**B**) The published literature on ocular DDSs for anterior segment diseases. (**C**) The published literature on PCO and anti-PCO DDSs. The literature was searched using the database of the Web of Science Core Collection from the year 2008 to 2022. The detailed search method is presented in Tables S1–S3 of the Supplementary Material.

The presence of ocular barriers (Figure 1A) is the major obstacle impeding effective drug dosing within the lens capsule. In the anterior segment, the cornea and conjunctiva physically limit drugs from entering the anterior segment of the eye, while the blood–aqueous barrier dynamically eliminates drugs from the aqueous humor. Ocular drug delivery dosage forms for the anterior segment of the eye have been gaining popularity due to innovations that allow them to overcome ocular barriers, control drug release, reduce toxicity and enhance intraocular drug bioavailability (Figure 1B) [15,16,17,18,19]. In particular, drug delivery systems (DDSs) for PCO have been thoroughly investigated during the past decade (Figure 1C). Although an ophthalmic DDS product for PCO eradication remains at the preclinical stage, several anti-inflammatory steroid-based slow-release DDSs have been clinically applied through various routes after cataract surgery, including intracameral injection (DEXYCU), anterior chamber insertion (Surodex) and intravitreal implantation (Ozurdex) [20,21,22]. For example, an anterior chamber dexamethasone (DEX) drug delivery suspension (DEXYCU) has been applied intracamerally to treat postoperative inflammation, and its single dose provides efficacious treatment for patients undergoing cataract surgery for up to 21 days [20].

This review focuses on ocular drug delivery to the anterior segment of eye with emphasis on controlled drug release kinetics to enable long-lasting therapeutic drug concentrations for PCO prevention post cataract surgery. We summarize and compare conventional and nanotechnology-based drug delivery approaches to PCO prophylaxis. Various examples of drug administration routes, fabrication techniques and therapeutic efficacy of those DDSs are provided, among which is the growing number of surface-modified IOL materials that serve as drug reservoirs to inhibit LECs and manage PCO formation. We have tabulated the release duration, release amount, and release half-life of anti-PCO DDSs presented in the literature and critically analyzed the drug release profiles. To design clinically translational prophylactic DDSs for PCO therapies, the issues of drug loading content, burst release and intraocular-environment-responsive release are highlighted. We discuss the design of long-acting ocular DDSs, delivery of drug combinations, and long-term ocular safety for improved patient compliance and quality of life.

## 2. Drug Delivery Approach to PCO Prophylaxis

### 2.1. Conventional Delivery of Free Drug(s) Solution

Various mechanisms of drug action have been explored to eliminate residual LECs inside the capsular bag. These include targeting different processes of PCO development, including anti-proliferation, anti-migration, anti-adhesion and anti-metabolite [12]. The intracapsular application methods of drug delivery for these purposes include direct injection, sealed-capsule irrigation (SCI) devices, and implants (i.e., lens refilling and IOL). Table 1 [23,24,25,26,27,28,29,30,31,32,33,34,35] summarizes those drugs, their targeting mechanisms and administration methods in both human and preclinical PCO animal models. The literature was selected based on two criteria: (1) the drug effect was evaluated in vivo and (2) the drug dose was provided. Distilled water is considered the only clinically safe agent to cause LEC lysis (via water-mediating hypoosmotic stress). To apply intracapsular distilled water, Zhang et al. utilized a fluid-air-dropping technique for which a syringe was connected to a silicone-tipped cannula for water loading and removal. Sterile air in the capsular bag prevented exposure of adjacent tissues in the anterior chamber to distilled water, thus allowing for selective targeting of LECs [23]. Surgical complications were not observed, nor was there any apparent damage to adjacent structures [23]. It is relevant to note a human trial study of the aldose reductase inhibitor, Sorbinil. This drug was administered orally (100 or 200 mg twice daily) or topically (0.5 mg) to diabetic patients undergoing intracapsular extraction [24]. In patients, Sorbinil was transported across the aqueous humor into the lens in humans, and a later study in mice revealed Sorbinil attenuated induction of α-SMA and E-cadherin, which are critical EMT marker proteins responsible for LEC migration and EMT during PCO formation [24,25]. 

Direct injection of single doses of potent drugs to the anterior and posterior chamber is a common method to kill LECs after phacoemulsification. Although the effectiveness of some drugs, such as mitomycin C and 5-Fluorouracil (5-Fu), in the inhibition of LEC proliferation has been identified, dose-associated ocular toxicity, such as corneal edema, remains a critical concern [26,30]. For example, to reduce the opacification of the capsular bag that is associated with lens replacement, 5 min treatment inside of the lens capsular bag with a solution of the two anti-proliferation drugs, actinomycin D and cycloheximide, reduced the development of visible capsular opacification for three months in rabbits; however, some of the animals displayed completely opaque cornea [30]. To reduce drug exposure to healthy ocular structures, a channel device SCI is applied intraoperatively to isolate the capsular bag in situ before drug treatment [36]. Kim et al. used SCI to compare the efficacy and toxicity of intraoperatively injected antiproliferative mitomycin C (0.04 mg/mL) and distilled water in rabbits after endocapsular phacoemulsification. The drug solution and SCI device were removed 2 min after injection. Compared to those administered distilled water, rabbits treated with SCI combining mitomycin C had a smaller opacification area in the posterior capsule and showed no toxicity in the surrounding ocular tissues [29].

The utilization of ocular implants, such as IOLs and lens refilling, is an alternative method for the application of free drugs into the capsular bag. Lens refilling has demonstrated a significant reduction in the PCO process [31,32,33]. In a rhesus monkey model, actinomycin-D was delivered into the capsular bag by application of the lens-filling material sodium hyaluronate (1%) before refilling with a silicone polymer [32]. An IOL can be pre-soaked in the drug solution before implantation for use as a vehicle of drug application. For example, implantation of an IOL coated with DEX was shown to reduce the inflammation post operation in rabbit eyes [34]. Duncan et al. chose a strongly hydrophobic poly-methylmethacrylate (PMMA)-based IOL for coating a hydrophobic drug, thapsigargin, an inhibitor of endoplasmic reticulum Ca^2+^ ATPase, by immersion. In a human lens capsular bag culture, thapsigargin was released, slowly reaching LECs and leading to inhibition of the growth in residual anterior LECs at drug concentrations as low as 200 nM [35]. However, the process of coating the IOL, such as the drying procedures, raised the concern of causing toxic anterior segment syndrome and blocking the visual axis [34].

**Table 1:** Conventional pharmacological approaches to PCO prophylaxis.

Reference	Drug Used	Treatment Dose and Length	Application Methods in the Capsular Bag (In Vivo Models)	Drug Action	Therapeutic Efficacy
[23]	Distilled water	0.1 mL for 3 min	Dropping water with a modified syringe using fluid/air exchange technique (Human)	Hypoosmotic stress	Damaged LECs from the anterior capsule without damage to intraocular structures.
[24]	Sorbinil	Oral: 200 mg or 400 mg q.d for 7 days; Topical: 0.5 mg up to 14 h	Oral and topical (Human)	Aldose reductase inhibitor (anti-oxidation)	Length of treatment was too short to effect lens sugar or sugar alcohol levels.
[25]		10 mg/kg	Intraperitoneal injection (Mice)		Inhibition of LEC EMT in mice.
[26]	Dexamethasone	Single dose: 4 mg/mL	Subconjunctival injection (Rabbit)	Anti-inflammation	Reduced LEC proliferation on the posterior capsule and effectively prevented PCO
	Diclofenac	Single dose: 2.5 mg/mL	Injection with an anterior chamber cannula (Rabbit)		
	RGD peptide ^a^	Single dose: 2.5 mg/mL		Anti-adhesion	
	EDTA ^a^	Single dose: 8 mg/mL			
	Mitomycin C	Single dose: 0.04 mg/mL		Antimetabolites	
[27]	N-Acetylcysteine	Single dose: 25 μL, 10 mmol/L	Injection into the eye chamber (Mouse)	Antioxidant	Attenuated LEC EMT signaling.
[28]	EDTA	1, 2.5, and 5 mg with a single dose	Intracameral injection (Rabbit)	MMP inhibitor	Reduced the degree of PCO by suppressing the matrix metalloproteinase activity.
[29]	Distilled water	/	Injection through SCI device (Rabbit)	Hypoosmotic stress	Reduced PCO development without toxicity to surrounding ocular tissues.
	Mitomycin C	0.4 mg/mL for 2 min		Antimetabolite	
[30]	Actinomycin D ^a^	10 μg/mL for 5 min	Flush with the Perfect Capsule Device and sodium hyaluronate (Rabbit)	Anti-proliferation	Reduced the formation of visible capsular opacification.
	Cycloheximide ^a^	25 μg/mL for 5 min			
[31]	Methotrexate ^a^	10 μM for 5 min	Human capsular rhexis specimens; Lens refilling (Rabbit)	Antimetabolite	Ablated viable LECs ex vivo, and delayed PCO formation in vivo.
	Actinomycin D ^a^	10 μM for 5 min		Anti-proliferation	
[32]	Sodium Hyaluronate	2.3% for 5 min	Lens refilling (Monkey)	Influencing LEC growing pattern	No capsular bag fibrosis.
[33]	Sodium Hyaluronate	1.4% for 3 min	Refilling (Rabbit)	Anti-proliferation	Distilled water and EDTA were most effective against PCO development.
	Balanced salt solution	/			
	Mitomycin C	0.2 mg/mL for 3 min			
	EDTA	10 and 15 mM for 3 min			
	5-Fluorouacil	33 mg/mL for 3 min			
	Acetic acid	3%, 0.3% and 0.003% for 3 min			
[34]	Dexamethasone	IOL incubated in 1 mg/mL	Implantation of IOL pre-soaked with the drug (Rabbit)	Anti-inflammation	Reduced postoperative inflammation.
[35]	Thapsigargin	IOL incubated in 0.2–2 μM	Insert into the capsular bag (Human)	Inhibitor of endoplasmic reticulum (Ca^2+^)-ATPase	Reduced LEC growth in the capsular bag.

^a^ The drugs were combined. Abbreviations: EDTA—ethylenediaminetetraacetic acid; EMT—epithelial–mesenchymal transition; MMP—matrix metalloproteinase; SCI—sealed capsular irrigation; RGD peptide—Arg-Gly-Asp tripeptide recognition sequence.

### 2.2. Nanotechnology-Based Drug Delivery

**Figure 2:**
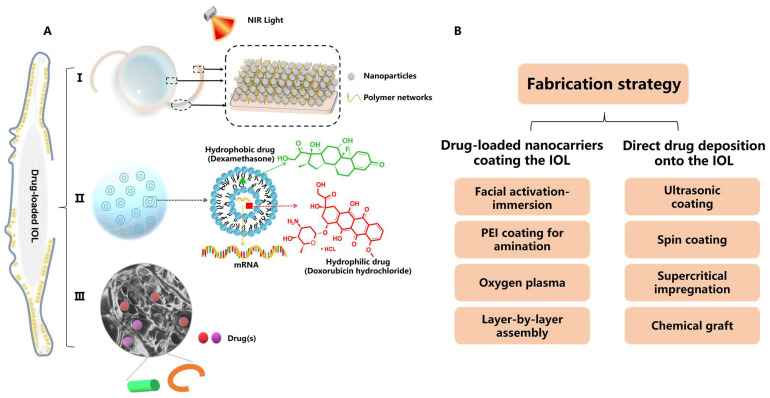
(**A**) Illustration of DDSs employing nanotechnology for PCO prevention, including I. drug-loaded IOL, II. NPs and their hydrogel composite and III. implants such as capsular tension ring and solid pellet/inserts. Inset: an example of polymeric nanostructure carrying pharmacological agent(s). (**B**) Fabrication strategies of drug-loaded IOLs for PCO therapy.

Various pre-clinical prophylactic DDSs have been developed over the past decade for PCO treatment, including I. drug-loaded IOLs, II. nanocarrier and hydrogel composites and III. implants (e.g., capsular tension ring, and solid pellet/inserts) (Figure 2A). Those DDSs can be designed for stimuli-responsive controlled drug release, such as external near-infrared (NIR) light, and for loading versatile drugs (e.g., anti-inflammatory drugs, antibiotics, chemotherapeutic drugs and mRNA for gene therapy). The data presented in Table 2 [37,38,39,40,41,42,43,44,45,46,47,48,49,50,51,52] were selected from the literature based on the following criteria: (1) pharmacological agents(s) were loaded within the DDS for PCO prevention and (2) data describing the measurement of drug release were available. Table 2 details the fabrication methods, drug loading and drug release of anti-PCO DDSs, in which IOLs modified for a drug reservoir have been extensively studied for post-cataract operative care. Based on the fabrication processes, anti-PCO DDS are classified into the following types: (1) drug-loaded nanocarriers coating the IOL; (2) direct drug deposition onto the IOL; (3) other dosage forms, including nanoparticles (NPs), implants and hydrogel composites. To control the pathological activities of LECs, all of these DDSs are capable of sustaining drug release for an extended period of time, while causing no toxicity to normal ocular tissues.

#### 2.2.1. Surface Modification of IOL Materials

An IOL-enabled DDS is an integrated object that is implanted along with the phacoemulsification. The design of a drug-loaded IOL involves coating the optical surface, rim, or haptics of the IOL with the drug-loaded nanocarriers and free drug. The specific fabrication strategies are outlined in Figure 2B. Most IOLs coated with drug-loaded nanocarriers are prepared with a single layer, using one of several different methods [37,38,39,40]. Mao et al. fabricated BP-DOX@IOL by integration of doxorubicin (DOX)-loaded black phosphorus nanosheets onto the non-optical section of the IOL via facial activation-immersion. BP-DOX@IOL exhibited a superior ability to inhibit PCO in vivo [37]. In another study, the IOL was surface-activated by oxygen plasma and further deposited with rapamycin (Rapa)-loaded Ti_3_C_2_ nanosheets (Rapa@Ti_3_C_2_) using spin-coating. Rapa@Ti_3_C_2_-IOL inhibited PCO for four weeks without obvious pathological damage in healthy ocular structures [38]. Nanomaterials applied in the above two examples (BP-DOX@IOL and Rapa@Ti_3_C_2_-IOL) were intrinsically photo-responsive, and their drug release was triggered by irradiation with near-infrared (NIR) light [37,38]. The IOL can also be activated by soaking the IOL overnight in an aqueous solution of polyethyleneimine (PEI) to generate a positively charged substrate surface before coating with NPs. For example, DOX@Exos-IOL was prepared via the immersion of a PEI-coated IOL in a DOX-loaded exosomes suspension. Exosomes as nanoscale extracellular vesicles have been used as a DDS due to their high biocompatibility, low toxicity and homologous targeting. In rabbits with phacoemulsification combined with IOL implantation, DOX@Exos-IOL was biocompatible and significantly eliminated LECs between the optical IOL and the anterior and posterior capsule [39]. Unlike the single-layer-coated IOL, Lin’s group developed a polysaccharide multilayer modified IOL [41]. In this case, the PEI-coated IOL was sequentially immersed in heparin solution and then chitosan NPs, followed by rinsing and drying to obtain an HEP/CTDNP-modified IOL via layer-by-layer deposition. At pH 5.5, which resembles the slightly acidic cellular microenvironment of the LECs, IOLs modified with HEP/CTDNP exhibited slow release of DOX and no burst release [41].

Pharmacological agents can be deposited directly onto the IOL via physical coating or chemical grafting [42,43,44,45,46,47]. For the drugs bromfenac and indomethacin, Yao’s group used ultrasonic spray technology to deposit a poly(lactic-co-glycolic acid) (PLGA) coating containing the drug onto the plate haptics of an IOL [42,43]. Compared to conventional coating systems, ultrasonic spray nozzles produced atomized PLGA drop sizes which allowed for a more precise and more controllable coating on the haptics of the IOL without damaging the optical part. The drug-loaded IOL containing either bromfenac or indomethacin displayed excellent anti-inflammatory and anti-PCO effects. Interestingly, despite using the same drug-loaded IOL, the drug release profiles of bromfenac and indomethacin were distinct. The release duration was 56 days for indomethacin versus 14 days for bromfenac, probably due to drug–PLGA interactions.

Spin-coating is another simple, reliable polymer coating technique for the preparation of thin films on substrates for drug deposition. Lu et al. developed the CsA@PLGA-IOL with a concentric annular coating for anti-inflammation post cataract surgery. The drug loading density and encapsulation efficiency of the drug cyclosporin A (CsA) were optimized by adjusting process parameters such as rotation speed, time duration and polymer concentrations. In LECs, CsA induced autophagic cell death, the key self-degradative cellular process. Intraocular implantation of a CsA@PLGA-IOL in rabbits prevented PCO formation without opacity on the central region; however, the optical resolution was influenced by the thicker peripheral ring coating areas in this particular ring-patterned coating of the IOL [44]. Supercritical impregnation is a new technology that does not require that organic solvents be used in the pre-soaking and spray-coating methods. In a recent study, to coat the IOL polymer with a lipophilic antimetabolite drug, methotrexate, the protected IOL was placed within a high-pressure cell and exposed to the drug dissolved in supercritical carbon dioxide (scCO_2_) [45]. scCO_2_ is recognized as a safe impregnation carrier. By varying the conditions, such as pressure and duration, different encapsulated amounts of methotrexate within the hydrophobic polymeric IOL support were achieved (i.e., 0.43–0.75 µg•mg^−1^ IOL). Finally, the drug can also be directly grafted onto the activated IOL via chemical reaction. For example, the PEI-coated IOL was immersed into a poly (PEGMA-co-GMA) (PPG) solution followed by immobilization of DOX on the surface of the PPG grafted substrate of IOL though the reaction of epoxy and amino groups. The multifunctional IOL surface modification exhibited sustained DOX release, inhibited adhesion and proliferation of LECs and reduced significantly the incidence of PCO in rabbit eyes [46].

#### 2.2.2. Development of Non-IOL Dosage Forms

Other nanotechnology-enabled dosage forms have been developed to adapt various administration routes, such as injectable formulations or implantable pellet/inserts [49,50,51,52]. A composite docetaxel (DTX) capsular tension ring (CTR) was fabricated via the polymerization of high internal phase emulsion (poly HIPES) of porous PMMA. The DTX-CTR not only enhanced the bending strength of materials made into the CTR for the capsular bag support, but it also effectively suppressed the occurrence of PCO for up to 6 weeks without damage to normal ocular tissues [49]. A PLGA-based, DEX-loaded implant pellet was manufactured using a bench-top pellet press. This implant system has a diameter of 2 mm and a thickness of about 1.5 mm and can be injected readily through standard small incision during cataract surgery. It achieved a long-acting anti-inflammation effect by releasing the drug for up to 42 days with near zero-order kinetics without signs of toxicity in vivo [51]. Hydrogel-based hybrid NP depots require intraocular administration via periocular or intracameral injections. For example, genistein (Gen)-loaded nanostructured lipid carrier synthesized by homogenization emulsification was subsequently mixed into a solution of two drugs, DEX and moxifloxacin (MOX), to prepare a temperature-sensitive in situ hydrogel (GenNLC-DEX-MOX hydrogel). GenNLC-DEX-MOX hydrogel exhibited differential drug release kinetics, leading to reduced inflammation, proliferation and myofibroblast transformation of the LECs in the process of PCO [50].

**Table 2:** Nanotechnology enabled drug delivery for PCO prophylaxis.

Drug-Loaded IOL								
Reference	Fabrication Method (Loaded Drug)	Part of IOL Modification	IOL Material	Drug Loading	Release Profile	Release Medium	Release Duration (t_50_ /t_90_ in Days)	Maximum Release%
[37]	IOL immersion into the BP-DOX solution via facial activation-immersion (Doxorubicin)	Non-optical	Hydrophobic acrylic	/	Photo-responsive	PBS (pH 7.4)	16 days (104/1148)	13%
[38]	Rapa@Ti_3_C_2_ was deposited onto the oxygen plasma-activated IOL with a spin-coater (Rapamycin)	Optical	Hydrophobic acrylic	/	NIR-triggered	Aqueous humor	2 days (0.43/2.9)	74%
[39]	DOX@Exos immobilized on the aminated IOL surface by electrostatic self-assembling (Doxorubicin)	Optical	Hydrophobic acrylic	/	A slow and continuous release	PBS (pH 7.4)	3 days (6.3/264)	40%
[40]	Fluorine ion beam-activating IOL was soaked in 5-Fu-CSNP suspension (5-Fluorouracil)	Optical	Hydrophobic PMMA	/	A burst release of the drug in 2 h followed by slow release	PBS (pH 7.2)	4 days (0.085/2.8)	100%
[41]	The activated IOL alternatively coated with heparin and drug-loaded NPs (CTDNPs by layer-by-layer assembly (Doxorubicin)	Optical	Hydrophobic acrylic	/	pH-responsive; no burst release	Acetate buffer (pH 5.5)	7 days (4.4 × 10^4^/9.6 × 10^7^)	7.2%
[42,43]	Drug-loaded PLGA was sprayed by ultrasonic coating system (Bromfenac or Indomethacin)	Plate haptics	Hydrophobic acrylic	0.1 mg	Biphasic release profiles	PBS	Bromfenac: 14 days (0.93/5.9) Indomethacin: 56 days (12/89)	Bromfenac: 91% Indomethacin: 80%
[44]	Drug-loaded PLGA coating by spin-coating (Cyclosporin A)	Thin center and thick periphery	Hydrophobic acrylic	/	Four-phase release: (1) exponential; (2) linear; (3) burst; (4) plateaued.	PBS (pH 7.4)	120 days (23/126)	78%
[45]	Supercritical impregnation (Methotrexate)	Optical	Hydrophobic acrylic	0.0069 mg	Sustained release	Aqueous humor (pH 7.2)	87 days (31/143)	>90%
[46]	The aminated IOL chemically grafted with PPG followed by DOX immobilization via the reaction of epoxy and amino groups (Doxorubicin)	Optical	Hydrophobic acrylic	/	pH-responsive	Sodium acetate buffer (pH 5.5)	7 days (4.8 × 10^4^/1.6 × 10^8^)	8.1% (pH 5.5)
[47]	Drug-loaded polydopamine coating followed by MPC immobilization via immersion (Doxorubicin)	Optical	Hydrophobic acrylic	/	A burst release of 75% of drug in the first 24 h followed by drug sustained release	Sodium acetate buffer (pH 5.5)	21 days (0.098/9.6)	>85% (PDA(DOX)-MPC)
[48]	Soaking IOLs in solution containing dual drugs (Moxifloxacin/ketorolac)	Optical	Hydrophobic G-free^®^ and hydrophilic CI26Y	/	An extended release of dual drugs for 26 days	PBS	26 days	CI26Y IOL: MOX: 52 μg ketorolac: 63 μg G-free^®^ IOL: MOX: 6 μg ketorolac: 7 μg
**Other DSS**								
**Reference**	**Drug Carrier**	**Fabrication Method**		**Drug Loading**	**Release Kinetics**	**Release Medium**	**Release Duration** **(t_50_ /t_90_ in Days)**	**Maximum Release%**
[49]	Capsular tension ring (DTX-CTR)	Porous PMMA via polyHIPE in combination with P(HEMA-co-MMA)-PMMA composite (Docetaxel)		/	Porous structure controlled sustained release	PBS (pH 7.4)	6 weeks	5.3 mg/g
[50]	Nanoparticle–hydrogel composite (GenNLC-DEX-MOX hydrogel)	NPs were mixed with the gel solution (Combination of Genistein Moxifloxacin and Dexamethasone)		Gen: 10 mg DEX: 4 mg/mL MOX: 2 mg/mL	Multiple drug release with differential kinetics	PBS (pH 7.0–7.6)	Gen: 40 days (20/127) DEX: 40 days (6.4/24) MOX: 10 days (1.8/7.4)	63% (Gen) 97% (DEX) 99% (MOX)
[51]	PLGA microparticles (DXM-PLGA)	Oil-in-water (*o*/*w*) emulsion-solvent extraction method followed by bench-top pellet press (Dexamethasone)		0.32 mg	Initial burst release followed by a sustained release	BSS (pH 7.4)	22 days (18/45)	53%
[52]	NPs (MePEG-PCL DOX NPs)	Solvent evaporation (Doxorubicin)		0.25 mg	Initial burst release followed by a sustained release of the drug	PBS (pH 7.4)	10 days (4.1/99)	75%

All DDSs were administered via implantation unless otherwise specified; reference [52] used subconjunctival injections. All the data reported for the maximum drug release were obtained from the papers’ release curves unless otherwise specified; the data point obtained by Getdata Software, and t_50_ and t_90_ were determined using DDsolver Software (see Supplementary Material Table S4). In references [45,46,47], the maximum drug release was calculated using the reported values of released drug amount divided by total drug loading amount. Abbreviations: BP-DOX—doxorubicin-loaded black phosphorus nanosheets; CI26Y—a hydrophilic IOL material, chemically crosslinked copolymer; CSNP—5-Fluorouracil-loaded chitosan nanoparticles; CTDNP—doxorubicin-incorporated chitosan nanoparticles were fabricated by sodium tripolyphosphate gelation; DOX@Exos—doxorubicin-loaded exosomes; DTX-CTR—docetaxel-loaded capsular tension ring; DXM-PLGA—dexamethasone-loaded PLGA microspheres; 5-Fu- PLGA—poly (lactic-co-glycolic acid); GenNLC-DEX-MOX hydrogel—temperature-sensitive drug delivery system carrying dexamethasone, moxifloxacin and genistein, nanostructured lipid carrier modified by mPEG-PLA based on F127/F68 as hydrogel; G-free^@^—a hydrophobic acrylic-based IOL material; MePEG-PCL DOX NPs—poly (ethylene glycol) methyl ether-block-poly (ε-caprolactone) doxorubicin-loaded nanoparticles; MPC—2-methacryloxyethyl phosphorylcholine; Rapa@Ti_3_C_2_—ultrathin Ti_3_C_2_ MXene nanosheet-coated IOL loaded with Rapamycin; PMMA—poly (methyl methacrylate); P(HEMA-co-MMA)-PMMA—copolymer of methyl methacrylate and 2-hydroxyethyl methacrylate combined with the polyHIPE to form a novel composite.

### 2.3. Pros and Cons of Conventional and Nanotechnology-Based Drug Delivery

**Figure 3:**
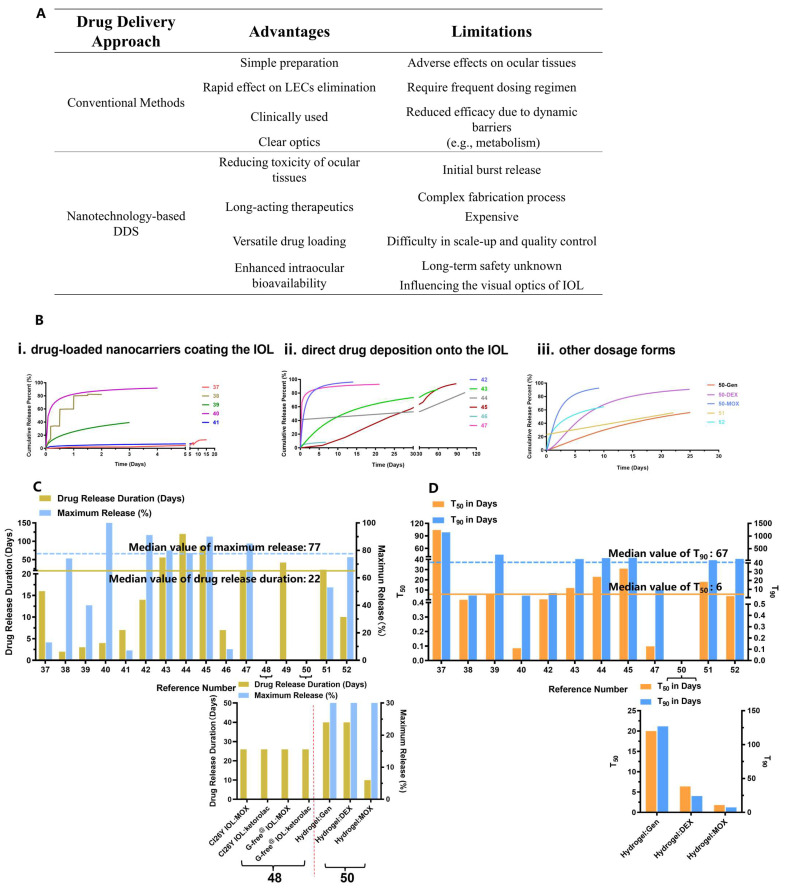
Analysis of prophylactic drug delivery strategies for PCO. (**A**) Advantages and limitations of conventional and nanotechnology-based drug delivery; (**B**) Fitted drug release profiles of anti-PCO DDSs shown in Table 2, including (**i**) drug-loaded NPs coating the IOL; (**ii**) direct drug deposition onto the IOL and (**iii**) non-IOL DDSs, including NPs, hydrogel and implants. The number shown on the X-axis corresponds to reference numbers in Table 2, and left- and right- y axis are drug release duration and maximum release percent, respectively; (**C**) Drug release duration and maximum drug release percent of nanotechnology-based DDS; (**D**) The time t_50_ and t_90_ when the fraction of drug release reached 50% and 90%, respectively, for nanotechnology-based DDSs. Note, (**i**) the data on “drug release duration” for references [48,49] were shown in Figure 3C, and not displayed in Figure 3B,D due to lack of data on drug release; (**ii**) the data on t_50_ and t_90_ for references [41,46] were too large to display in the graph in Figure 3D, and references [48,49] did not provide sufficient data for calculating t_50_ and t_90_. (**iii**) X-axes of the two graphs in Figure 3C,D represent the data on drug combination.

Both conventional and nanotechnology-based drug delivery can eliminate residual LECs to some extent, but each delivery method has its own advantages and limitations (Figure 3A). The conventional drug delivery shown in Table 1 is often assisted by mini devices (e.g., SCI) to isolate the capsular bag for direct administration of a high dose (i.e., milligrams) of chemotherapeutic drugs. Despite its inhibition of PCO formation in animal models and reported potency in clinical applications, the use of conventional delivery of free drugs in the clinic is limited, due to the adverse effects on surrounding healthy ocular structures. In addition, a drug administered intraocularly may be eliminated quickly owing to (1) the rapid turnover of aqueous humor (90–100 min) via the trabecular meshwork and Schlemm’s canal and (2) transport through the blood–aqueous barrier into blood circulation [53,54]. Thus, direct administration does not provide a long-acting control of PCO development.

The value of novel nanotechnology-assisted pharmacological interventions in concert with optimal IOLs is increasingly recognized by clinicians [55]. As shown in Table 2, currently, most dosage forms are drug-loaded IOLs, which are implanted into the capsular bag during cataract surgery. We used the DDsolver program for modeling and comparison of drug release profiles of (i) drug-loaded NPs coating the IOL, (ii) direct drug deposition onto the IOL and (iii) other dosage forms (i.e., NPs, hydrogel, implants) (Figure 3B) [56]. It was found that anti-PCO DDSs studied in Table 2 exhibited various drug release profiles, and the prolonged drug release duration and maximum drug release percent were found to be 22 days and 77%, respectively (Figure 3C). Further analysis of the fitted drug release profiles revealed that the median values for 50% (t_50_) and 90% (t_90_) drug release were about 6 days and 67 days, respectively (Figure 3D). Sustained drug release over a protracted period can improve drug potency against LECs. For example, IOLs modified with 5-Fu chitosan nanoparticles (Nano-5-Fu-IOL) exhibited the half inhibition dose of 0.2 μg/mL against human LECs compared to 1 μg/mL for the free drug solution [40]. In vivo, direct implantation of Nano-5-Fu-IOL at a dose of 5-Fu equal to 19.5 mg (0.2 mL, 97.8 mg/mL) markedly inhibited the occurrence of PCO without inflammation in rabbit eyes. On the other hand, direct injection of a high dose of a 5-Fu solution (33 mg/mL) was ineffective in preventing anterior capsule proliferation [33,40]. These data indicate that the DDS can lower the therapeutic dose by a hundred- or thousand-fold which, in turn, leads to reduced adverse effects.

## 3. Design Consideration of Anti-PCO DDS

### 3.1. Long-Acting DDS

Although nanotechnology-based DDS is promising for long-term PCO prevention, nearly all dosage forms are still in the developmental stage, with translational challenges for controlled drug delivery and ocular safety [57]. Chronic drug administration with a DDS to the anterior chamber is of particular interest for the clinical control of the pathological transformation of LECs, owing to the long period of PCO development, typically several years after cataract surgery. In the following sections, we discuss three design aspects of long-acting ocular drug delivery for anti-PCO therapy, including (1) stimuli-responsive drug release, (2) initial burst release, and (3) drug loading content.

#### 3.1.1. Exploitation of the Intraocular Environment for Stimuli-Responsive Release

**Figure 4:**
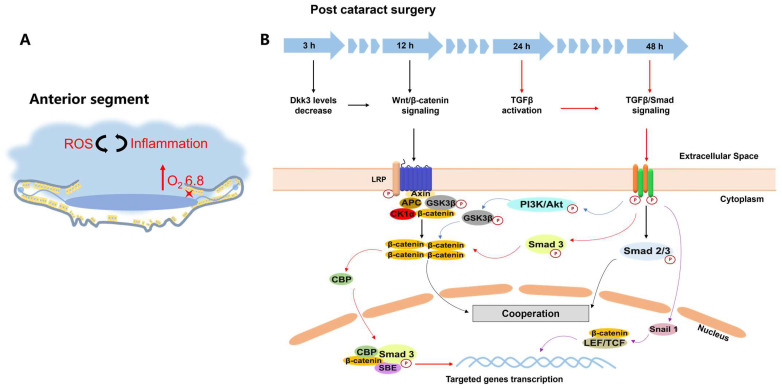
Complex pathological processes during PCO formation post cataract surgery. (**A**) Cataract surgery alters the intraocular environment. The level of oxygen (pO_2_) increases greatly, anterior to the lens near the trabecular meshwork. The antioxidants (e.g., glutathione and ascorbic acid) are rapidly depleted, which is then accompanied by increased oxidative stress and accumulation of pro-inflammatory cytokines (e.g., TGFβ). (**B**) Temporal regulation of both canonical Wnt/β-catenin and TGFβ/Smad signaling involved in cell proliferation and EMT after cataract surgery at early and later stages, respectively. *Dickkopfs* (Dkk) 3 protein levels fall sharply as early as 3 h upon removal of lens fiber cells, and Wnt reporter activity upregulates in remnant LECs by 12 h. Simultaneously, accumulated TGFβ is activated within 24 h, and canonical TGFβ signaling is activated in LECs at about 48 h. Note, the symbol 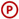 represents Ser/Thr phosphorylation, where the signaling pathways are activated upon phosphorylation.

Cataract surgery causes abrupt changes to the intraocular environment; these include changes in pH, the concentration of cytokines, oxygen distribution, redox status, etc. (Figure 4A). Cataract surgery increases the level of oxygen (pO_2_) (i.e., 3.5 to 6.8 mmHg) in the anterior chamber angle [58]. Rapid depletion of glutathione and ascorbic acid leads to increased concentrations of reactive oxygen species (ROS), including hydrogen peroxide (H_2_O_2_), as well as hydroxyl (^•^OH), and superoxide (O_2_^•−^) radicals [58,59,60]. LECs produce an array of pro-inflammatory cytokines in the aqueous humors that can activate molecular signaling (e.g., Wnt/β-catenin, TGFβ) associated with EMT of LECs [61]. Thus, PCO prevention may be achieved by exploitation of biocompatible materials that are susceptible to such endogenous chemical and biological triggers to direct the spatial and temporal release of drugs.

Ophthalmic DDSs based on stimuli-responsive polymers have been designed for dose-controlled release [62,63]. To exploit the pathological pH during PCO development, Lin’s group designed pH-responsive drug-loaded IOLs using two different surface modifications [41,46]. One was prepared via the layer-by-layer method to obtain an IOL coated with NPs of DOX-loaded multilayers and the polyelectrolyte chitosan (CTDNPs) [41], and the other was an IOL chemically modified with a single-layered binary copolymer grafted with DOX [46]. In vitro, both drug-loaded IOLs showed sustained drug release under pathological conditions (pH 5.5) for 7 days. In vivo, PCO was not found in rabbits implanted with the drug-eluting IOLs up to 2 months post operation. The underlying mechanisms of pH-responsive drug-loaded IOLs involve the protonation of the applied materials, such as chitosan, leading to the structural transformation and solubility changes of drug carriers. For example, CTDNP gel formed by the multilayer coating using chemical cross-linking was protonated and became soluble at pH 5.5, leading to the release of DOX. On the other hand, regardless of fabrication method, both pH-responsive drug-eluting IOLs reached a drug maximum release of less than 10%, raising the concern that the DDS is not sufficiently robust to rely solely on the pH stimulus for controlled drug release.

Nanomaterials with intrinsic therapeutic activities have also been exploited to activate drug release while exerting bioactivities, such as oxidation and anti-inflammation [64]. In PCO therapy, those bioactive nanomaterials are used to prepare NPs, and their LEC-killing effect and drug release are simultaneously triggered by exogenous stimuli, such as NIR and ultraviolet B [37,38,65,66]. In particular, NIR is a non-invasive, controllable, and stable stimulus compared to the varying levels of endogenous stimuli (e.g., pH, ROS), which depend on the pathological conditions. Irradiation with NIR can precisely tune the drug release rate on-demand via the frequency and duration of irritation. As a result, this approach can be helpful for temporal control of the biological events occurring at various stages of PCO development after cataract surgery (Figure 4B). For example, black phosphorus and Ti_3_C_2_ as photothermal materials were selected for the surface modification of IOLs [37,38]. At pH 7.4, the release of DOX or Rapa from NIR-responsive-material-coated IOL was promoted by irradiation with 808 nm laser light. DOX release from BP-DOX@IOL was triggered by exposure to 808 nm light for 3 min. Rapa release from Rapa@Ti3C2-IOL reached 74.1% by stimulating the DDS multiple times for 10 min at regular intervals, resulting in inhibition of LEC growth. 

#### 3.1.2. Issues of Burst Drug Release

The burst release of a drug from a DDS at the initial stage can both negatively and positively impact the effectiveness of PCO therapy. As shown in Figure 3B–D, many anti-PCO DDSs exhibited biphasic release profiles with a high initial rate of delivery followed by slow and stable drug release over a long period. The drug burst can be a desirable trait in the situation of delivering antibiotics (e.g., MOX) post cataract surgery to quickly inhibit bacterial pathogens that cause eye infection. Burst release is also commonly observed in stimuli-triggered release (e.g., pH or NIR) for controlled drug delivery under pathological conditions, as mentioned in Section 3.1.1. On the other hand, the burst release of a DDS can lead to negative consequences, such as local ocular toxicity due to high drug concentration, rapid drug clearance, drug waste, and a shortened release profile [67]. Especially for DDSs with ocular implants, the surge in drug concentration within the confined volume of the anterior chamber (i.e., 250 μL) may induce adverse effects of chemotherapeutic drugs that have a narrow therapeutic window [68]. For instance, release of nearly 30% (5.8 mg) of 5-Fu from an IOL modified with chitosan NPs (5-Fu-CSNP) within 2 h was associated with mild anterior chamber inflammation observed in three out of five animals. The burst release could compromise the effective lifetime of long-acting DDS for PCO therapy. The drug release of 5-Fu in the example given above was completed within 4 days, which was too short to prevent PCO development [40]. Even though drug release can be controlled through a stimuli-responsive mechanism, it is technically difficult to control the amount of initial burst release. This is illustrated by the pH-responsive IOL surface chemically modified by DOX immobilization, for which the burst release was similar at pH 7.4 and pH 5.5 [46]. Burst release may also cause toxicity at the site of injection. For example, the initial release of DOX (~10%) from nano-formulation injected via the subconjunctival route was potentially associated with scleral and corneal toxicities, although the slow release observed during the late phase decreased the drug efflux of DOX in LECs for improved intraocular concentration [52].

To reduce burst release, a zero-order DDS that exhibits a constant drug release rate is preferable, due to its ability to maintain stable drug concentrations over an extended period of time, thereby reducing frequent dosing and minimizing adverse effects [69]. Several anti-PCO DDSs have attained near zero-order release kinetics by new fabrication techniques (e.g., spin-coating) [44], surface modification of drug-layer-coated IOLs [47], or by using a blend of excipients [51]. Lu et al. applied spin-coating to fabricate centrifugally concentric, ring-patterned, cyclosporin A-loaded PLGA-coated IOLs (CsA@PLGA IOL). The release profile of CsA@PLGA IOL was linear for up to 8 weeks [44]. The burst release rate was minimized by increasing PLGA concentration (i.e., 10–50 mg/mL), and the duration of initial drug release was shortened by decreasing the drug loading content (i.e., 5 to 2.5 mg/mL). The authors hypothesized that the observed exponential drug release during the first week may be caused by drug adhesion to the outer surface of the polymeric thin film formed by spin-coating. It was suggested that the proper blend of PLGA and another polymer (i.e., PCL) may form desirable structural nanopores that would serve as drug reservoirs to control drug release [70]. The drug-loaded coating layer of an IOL surface can be further chemically modified to reduce the burst release. In the study of a polydopamine-coated IOL incorporating DOX, modification of its surface with 2-methacryloyloxyethyl phosphorylcholine (MPC) reduced DOX release by nearly 30% compared to the one without MPC, resulting in drug retention in the coating for sustained release [47]. Lastly, the use of blended excipients is an alternative strategy to modify the drug release curve for zero-order kinetics. For example, Chennamaneni et al. developed a bioerodible DEX implant based on PLGA for postoperative cataract inflammation. Compared to pure PLGA microparticles, addition of 10% hydroxypropyl methylcellulose (HPMC) altered the drug release profile close to zero-order kinetics despite a slightly higher burst effect [51]. More importantly, the authors correlated intraocular pharmacokinetics (PK) of their microparticle implant with a sustained release of the drug, and found that the exposure of DEX concentrations in aqueous and vitreous humor were dose-dependent as a result of the zero-order kinetics that kept drug release rate relatively stable over time [51].

#### 3.1.3. Determination of Drug Loading Content

Most of the publications cited in Table 2 did not report the drug loading content nor its calculation. Yet, knowing drug loading content is critical for chronic therapy, as it determines required drug dosing of the DDS in vivo [71]. Owing to drug clearance in the anterior segment (Figure 1A), to maintain the minimum therapeutic drug level (*C_ss,av_*) of a certain drug, the drug dose (*D*) loading into the DDS can be estimated using the following equation:

D=Css,av×CLAH×τF

where *C_ss,av_* is the average steady-state concentration, *CL_AH_* is the drug clearance in aqueous humor, *τ* is the dosing interval (e.g., days or weeks), and *F* is the available drug fraction in the anterior chamber [71]. We take DEX, a corticosteroid anti-inflammatory drug used clinically post cataract surgery, as an example to calculate its required dose for chronic administration in both (1) implantable and (2) injectable formulations:

(1) For implantable formulation, *F* = 1, *C_ss,av_* = 1.98 µg/mL, *CL_AH_* = 10 µL/min, *τ* = 28 days according to the literature [71,72].

The estimated required dose of DEX for an implant-based DDS is then calculated as:

*D* = 1.98 × 10 × 28/1 = 550 μg

In the study of DEX-loaded polymeric microparticle implant in rabbits, the target drug load of 20% was capable of sustaining a high dose (600 μg) of DEX in vivo, reaching therapeutic concentrations as high as 1.5 µg/mL in both aqueous and vitreous humors over 28 days [49].

(2) For the injectable formulation via conjunctival route, *F* = 0.05 ~ 0.1, *C_ss,av_* = 1.98 µg/mL, *CL_AH_* = 10 µL/min, *τ* = 28 days according to the literature [71,72].

The estimated required dose of DEX for an injectable DDS is then calculated as:

*D* = 1.98 × 10 × 28/0.1 or 1.98 × 10 × 28/0.05 = ~5500 μg ~ 11,000 μg

As such, the DEX concentration encapsulated in the hydrogel was 4 mg/mL [50]. Considering that the injection volume is less than 1 mL in the eye, the actual dose administered was far lower than the required dose, which may lead to quick drug exhaustion of the DDS in vivo.

To achieve high drug loading (>10 wt%) for long-acting DDS, the drug can be loaded via either a post-loading or co-loading processes before coating onto the IOL [73]. Post-loading of the drug involves synthetized nanocarriers as the first step, followed by mixing with a drug solution to obtain drug-loaded nanocarriers. For example, Ti_3_C_2_ MXene and BP nanosheets have achieved high loading of DOX and Rapa up to 847% and 92%, respectively [37,38]. The negatively charged nanosheets could load positively charged drugs through electrostatic interaction. In another study, LEC-derived exosomes were mixed with DOX and then electroporated, leading to 72% load capacity of DOX [39]. Secondly, co-loading of the drug involves the processes of simultaneously mixing drugs and polymers to form the composite drug reservoirs. For example, loading docetaxel onto the CTR implant for sustained drug release was a two-step process [49]. A porous PMMA framework with high porosity (74~95 vol%) was prepared with a high internal phase emulsion template, and then it was filled with the mixture of the drug docetaxel and copolymer of methyl methacrylate (MMA) and 2-hydroxyethyl methacrylate (HEMA) to form a composite. The CTR released docetaxel for up to 6 weeks in vitro and maintained effective drug concentration in the aqueous humor after 42 days.

### 3.2. Delivery of Drug Combination

The use of a combination of medications that target different pathologic processes is common practice in medicine, including ophthalmology [74]. A practical example of this is OMIDRIA, a combination medication containing ketorolac, tromethamine and phenylephrine that surgeons routinely inject during cataract surgery for pain relief and anti-inflammation [75]. In the treatment of retinal disease, polycaprolactone, as a highly biocompatible and biodegradable material, was used to co-encapsulate resveratrol and metformin for persistent drug release, which in turn led to enhanced retinal permeability and collective pharmacological activities, including antioxidation and anti-inflammation and angiogenesis [76]. Because PCO is a multifactorial eye disease involving the spatiotemporal coordination of various molecular pathways, such as TGFβ and Wnt/β-catenin signaling [61,77,78,79] (Figure 4B), selection of drug combination is a compelling prophylaxis for capsular opacification. In a pre-clinical study, different drug combinations with various drug actions have been applied to the capsular bag in rabbit eyes, and in all cases, poly-therapies demonstrated better efficacy than therapies with a single drug [26,30,31]. For example, actinomycin D and cycloheximide have different mechanisms of action: the former inhibits RNA synthesis, and the later inhibits protein synthesis. Microscopic analysis of the capsular bag treated with the combination of actinomycin D and cycloheximide had the least fibrosis compared to each drug alone [30]. In another study, the antibiotic MOX and the anti-inflammatory ketorolac were co-loaded onto the IOL. Both therapeutic concentrations of MOX and ketorolac were higher than single eye-drop treatments and were maintained for at least 7 days, leading to reduction in LEC adhesion on the IOL [48]. Furthermore, many cytotoxic agents used for LEC inhibition are chemotherapeutic drugs, and their combination may exhibit a synergistic effect, leading to improved drug potency and reduced drug toxicity [80]. This can be seen with DOX and MMC, two cytotoxic drugs applied in PCO therapy (Table 1 and Table 2). Though DOX–MMC combination has not yet been investigated against LECs, their synergistic interaction was shown in cancer cells at an extremely low doses (i.e., nanomolar) in contrast to single free DOX or MMC [81,82], indicating their potential application in PCO therapy.

Despite its effectiveness, combination therapy also may lead to unspecific tissue toxicity and sometimes antagonism in vivo due to different pharmacokinetics of each drug component. Although nanotechnology-assisted loading of drug combinations allows the delivery of desirable amounts or ratios of drug combinations [80], translation of multidrug DDSs remains a challenge. One of the major obstacles is the long-term coordination of drug release. To simultaneously prevent complications (e.g., infection, inflammation, and PCO) post cataract surgery and to improve patient compliance, DEX, MOX and Gen were co-loaded onto different structures of hydrogel and NP composite. To build such a DDS, the water insoluble Gen was first loaded into NPs (GenNLCs) by homogenization emulsification, and then DEX and MOX and pre-formed GenNLCs were mixed with a temperature-sensitive in situ hydrogel [50]. The resulting GenNLC-DEX-MOX exhibited multiple drug release with differential kinetics, in which MOX showed a quick and nearly complete release for up to 10 days, DEX was released at a constant rate for 7 days, whereas Gen was released slowly from NPs with the cumulative release of 63.4% at day 40.

### 3.3. Long-Term Ocular Safety

The safety assessment of ocular long-acting DDSs remains largely unexplored [83]. As a common carrier, IOL management as an anti-PCO DDS is an interesting and important issue in future clinical translation, mainly including non-toxicity of periocular tissues and non-obstruction of vision. A chronically administered DDS intentionally maximizes local drug exposure and reduces the dosing frequency via various routes of administration (e.g., implantation or injection). Those intraocularly retained DDSs may increase toxic concentrations of pharmacological agents and excipients and can elicit inflammatory and foreign-body responses. Presently, pre-clinical data gleaned from anti-PCO therapy focuses on global eye health, with an attention to corneal tissue toxicity, which can be evaluated with an ocular irritation test (e.g., Draize test), slit lamp bio-microscopy of eyeballs, corneal endothelial cell density, H&E staining histology, and surface intraocular pressure. However, those ocular safety evaluations only provide a roadmap of the basic toxic profiles of those chronically administered DDSs. Particularly, the modified IOL and CTR as a drug reservoir may elicit an accumulation and fusion of macrophages around the IOL and macrophage breakdown, consequently leading to interference of visual acuity [84,85].

Another safety issue is the fabrication processes of surface-modified IOLs, which could potentially influence their biocompatibility and functionality. As shown in Table 2, most anti-PCO DDSs used the IOL as the drug reservoir; as such, either the optical or the non-optical (e.g., plate haptics) region of the IOL must be pre-treated to load drugs. Despite careful evaluation of surface and optical properties of IOLs, such as surface wettability and morphology and light transmittance, the extent of IOL alteration by such physical or chemical treatments requires further investigation. For example, soaking the IOL in PEI aqueous solution is a common procedure to obtain an activated IOL with a negatively charged surface for drug loading [46]. However, it is found that the thickness of the charged coating of the hydrophobic acrylic IOL was positively corelated to the soaking time in PEI solutions [86]. The longer soaking time appeared to cause the charged coating of the IOL surface to become too thick to remain completely transparent. Because of these safety concerns, functions of both the IOL and DDSs coated onto IOLs must be validated prior to commercialization.

## 4. Conclusions

PCO eradication based on pharmacological measures is an expanding area of drug delivery. Nanotechnology-based ocular DDS renders long-acting drug therapy possible, and pre-clinical studies provide valuable insights into designing various drug reservoirs (e.g., IOL, hydrogels, NPs) through different administration routes. Consideration of criteria such as intraocular pharmacokinetics and barriers, the anterior environment for controlled stimuli-triggered drug release, zero-order release kinetics and sufficient drug loading are important for maintaining intraocular therapeutic concentrations in vivo for extended periods of time. Future in-depth investigation of ocular safety for those long-acting DDSs is needed for clinically translatable anti-PCO therapy.

## Abbreviation

5-Fu: 5-Fluorouracil; CTR: capsular tension ring; DDS: drug delivery systems; DEX: dexamethasone; DOX: doxorubicin; EMT: epithelial–mesenchymal transition; Gen: genistein; IOL: intraocular lens; LECs: lens epithelial cells; MOX: moxifloxacin; NIR: near-infrared; NPs: nanoparticles; PEI: polyethyleneimine; PCO: posterior capsule opacification; PLGA: poly (lactic-co-glycolic acid); PK: pharmacokinetics; PMMA: poly-methylmethacrylate; Rapa: rapamycin; SCI: sealed capsule irritation.

## Data Availability

The datasets analyzed during the current study are available from the corresponding author upon reasonable request.

## Supplementary Materials

The following supporting information can be downloaded at: https://www.mdpi.com/article/10.3390/pharmaceutics15041235/s1, Table S1. Query search of published literatures for ocular drug delivery system for anterior segment diseases in Web of Science Core Collection. Table S2. Query search of published literatures for posterior capsule opacification (PCO) in Web of Science Core Collection. Table S3. Query search of published literatures for drug delivery system for PCO in Web of Science Core Collection. Table S4. The goodness of fit of the model for drug release profiles of anti-PCO DDS.
