# Supplementary material for: Clinical Translation of Long-Acting Drug Delivery Systems for Posterior Capsule Opacification Prophylaxis
Source: Pharmaceutics. 2023 Apr 13;15(4):1235. doi: 10.3390/pharmaceutics15041235 (PMC10143098; doi:10.3390/pharmaceutics15041235)
Supplement: Supplementary file 1 [file pharmaceutics-15-01235-s001.zip › pharmaceutics-2322252-supplementary.pdf]

Supplementary Materials

Clinical Translation of Long-Acting Drug Delivery Systems for Posterior Capsule Opacification Prophylaxis

Xinyang Li [1], Chen Liang [1], Yexuan Guo [2], Jing Su [2], Xi Chen [1], Robert B. Macgregor, Jr. [3], Rui Xue Zhang [2,*] and Hong Yan [1,2,*]

[1] Xi'an People's Hospital (Xi'an Fourth Hospital), Shaanxi Eye Hospital, Affiliated People's Hospital of Northwest University, 21 Jiefang Road, Xi'an 710004, China
[2] Xi'an Key Laboratory of Stem Cell and Regenerative Medicine, Institute of Medical Research, Northwestern Polytechnical University, 127 West Youyi Road, Xi'an 710072, China
[3] Department of Pharmaceutical Sciences, Leslie Dan Faculty of Pharmacy, University of Toronto, 144 College Street, Toronto, ON M5S 3M2, Canada
* Correspondence: zhangruixue@nwpu.edu.cn (R.X.Z.); yan2128ts@med.nwu.edu.cn (H.Y.); Tel.: +86-17792282109 (R.X.Z. & H.Y.); Fax: +86-029-88460875 (R.X.Z. & H.Y.)

Table S1. Query search of published literatures for ocular drug delivery system for anterior segment diseases in Web of Science Core Collection

Searching Step	Searching Topic [a]
1	nanomedicine
2	nanoparticle
3	nanodevice
4	drug delivery system
5	intraocular lens
6	IOL
7	combine 1 OR 2 OR 3 OR 4 OR 5 OR 6
8	publication date range "2008-2022"
9	document types as "article" and "review article"
10	anterior segment disease
11	cataract-related disorder
12	cataract
13	corneal disease
14	combine 10 OR 11 OR 12 OR 13

15	publication date range “2008-2022”
16	document types as “article” and “review article”
17	combine 9 AND 16

[a] Topic term within a record include title, abstract, author keywords, keywords Plus®

Table S2. Query search of published literatures for posterior capsule opacification (PCO) in Web of Science Core Collection.

Searching Step	Searching Topic [a]
1	posterior capsular opacification
2	posterior capsule opacification
3	PCO
4	combine 1 OR 2 OR 3
5	publication date range “2008-2022”
6	document types as “article” and “review article”

[a] Topic term within a record include title, abstract, author keywords, keywords Plus®

Table S3. Query search of published literatures for drug delivery system for PCO in Web of Science Core Collection

Searching Step	Searching Topic [a]
1	posterior capsular opacification
2	posterior capsule opacification
3	PCO
4	combine 1 OR 2 OR 3
5	publication date range “2008-2022”
6	document types as “article” and “review article”
7	nanomedicine
8	nanoparticle
9	nanodevice

10	drug delivery system
11	intraocular lens
12	IOL
13	combine 7 OR 8 OR 9 OR 10 OR 11 OR 12
14	publication date range "2008-2022"
15	document types as "article" and "review article"
16	combine 6 AND 15

[a] Topic term within a record include title, abstract, author keywords, keywords Plus®

The drug release profile of DDS in each reference (**Table S4**) was selected if it was applied *in vivo*. Firstly, individual data points corresponding to the drug release cumulative time (t) in *x-axis* and the fraction (%) of drug released in time t (F) in *y-axis* were acquired by Getdata software. To assess the goodness of fit of a model for fitting drug release profiles, statistical criteria including the correlation coefficient (R^2), the coefficient of determination ($R^2_{adjusted}$), the mean square error (MSE), the Akaike Information Criterion (AIC) and the Model Selection Criterion (MSC) were determined by the program DDSolver [1]. Mathematical models used to fit the drug release profiles include the zero-order with T_{lag}, Logistic 1, Logistic 2 and Gompertz 1. The equations were as follows:

$$\text{Zero-order with } T_{lag}\text{: } F = k_0 \cdot \left(t - T_{lag}\right);$$

$$\text{Logistic 1: } F = 100 \cdot \frac{e^{\alpha+\beta\cdot\log t}}{1+e^{\alpha+\beta\cdot\log t}};$$

$$\text{Logistic 2: } F = F_{max} \cdot \frac{e^{\alpha+\beta\cdot\log t}}{1+e^{\alpha+\beta\cdot\log t}};$$

$$\text{Gompertz 1: } F = 100 \cdot e^{-\alpha\cdot e^{-\beta\cdot\log t}}$$

Where T_{lag} was the lag time prior to drug release, k_0 was the zero-order release constant, α was the scale factor in Logistic 1 and 2, Gompertz 1 and 2 models, β was the shape factor in Logistic 1 and 2, Gompertz 1 and 2 models, F_{max} was the maximum fraction of the drug released at infinite time.

After selecting the optimal drug dissolution model, the time (T_{50} and T_{90}) when F reached 50% and 90% were calculated, respectively. The unit was uniformly converted to days. The selected drug dissolution models and statistical criteria are shown in **Table S4**.

Table S4. The goodness of fit of the model for drug release profiles of anti-PCO DDS

**References** [a]	**Drug Dissolution Models**	**Statistical Criteria**
[2]	Logistic 1	R^2 0.9432; $R^2_{adjusted}$ 0.9337; MSE: 1.4910; AIC: 21.5295; MSC: 2.3683
[3]	/	/
[4]	Logistic 1	R^2 0.9884; $R^2_{adjusted}$ 0.9873; MSE: 1.9926; AIC: 39.9040; MSC: 4.1269
[5]	Logistic 1	R^2: 0.9822; $R^2_{adjusted}$: 0.9804; MSE: 17.9234; AIC: 66.2643; MSC: 3.6946
[6]	Gompertz 1	R^2: 0.9584; $R^2_{adjusted}$: 0.9584; MSE: 0.2248; AIC: 15.7712; MSC: 2.8722
[7]	Logistic 1	R^2: 0.9905; $R^2_{adjusted}$: 0.9893; MSE: 12.5206; AIC: 50.0682; MSC: 4.2536
[8]	Logistic 2	R^2: 0.9903; $R^2_{adjusted}$: 0.9871; MSE: 11.6764; AIC: 44.2439; MSC: 3.9695
[9]	Zero-order with T_{lag}	R^2: 0.9677; $R^2_{adjusted}$: 0.9657; MSE: 5.9742; AIC: 86.0807; MSC: 3.2119
[10]	Logistic 1	R^2: 0.9458; $R^2_{adjusted}$: 0.9439; MSE: 74.8207; AIC: 233.4190; MSC: 2.7824
[11]	Gompertz 1	R^2: 0.9837; $R^2_{adjusted}$: 0.9822; MSE: 0.0980; AIC: 4.9776; MSC: 3.8068
[12]	Gompertz 1	R^2: 0.9459; $R^2_{adjusted}$: 0.9351; MSE: 18.2460; AIC: 35.5937; MSC: 2.3461
[13]	/	/
[14]	/	/
[15]	Zero-order with T_{lag}	R^2: 0.9639; $R^2_{adjusted}$: 0.9518; MSE: 8.0458; AIC: 19.9188; MSC: 2.5201

[16]	Logistic 2	Moxifloxacin:<br>R^2: 0.9969; $R^2_{adjusted}$: 0.9962; MSE: 4.9608;<br>AIC: 46.4910; MSC: 5.2411
	Logistic 1	Dexamethasone:<br>R^2: 0.9904; $R^2_{adjusted}$: 0.9897; MSE: 10.9108; AIC:<br>78.3205; MSC: 4.3835
	Logistic 1	Genistein:<br>R^2: 0.9986; $R^2_{adjusted}$: 0.9968; MSE: 1.0821;<br>AIC: 43.6580; MSC: 5.5412
[17]	Logistic 2	R^2: 0.9425; $R^2_{adjusted}$: 0.9137; MSE: 54.2423; AIC:<br>43.6583; MSC: 1.9988

[a] Reference [3]: the drug release profile was pperiodically triggered by the NIR and exhibited the stair shape. The model was not available in DDSolver; references[13] and [14]: the drug release F cannot be obtained due to insufficient information of drug loading content.

References

1. Zhang, Y.; Huo, M.; Zhou, J.; Zou, A.; Li, W.; Yao, C.; Xie, S. DDSolver: an add-in program for modeling and comparison of drug dissolution profiles. *AAPS J* **2010**, *12*, 263-271, doi:10.1208/s12248-010-9185-1.

2. Mao, Y.Y.; Li, M.; Wang, J.D.; Wang, K.J.; Zhang, J.S.; Chen, S.Y.; Liu, X.; Liang, Q.F.; Gao, F.; Wan, X.H. NIR-triggered drug delivery system for chemo-photothermal therapy of posterior capsule opacification. *J Control Release* **2021**, *339*, 391-402, doi:10.1016/j.jconrel.2021.09.030.

3. Ye, Z.; Huang, Y.; Li, J.; Ma, T.; Gao, L.; Hu, H.; He, Q.; Jin, H.; Li, Z. Two-dimensional ultrathin Ti(3)C(2) MXene nanosheets coated intraocular lens for synergistic photothermal and NIR-controllable rapamycin releasing therapy against posterior capsule opacification. *Front Bioeng Biotechnol* **2022**, *10*, 989099, doi:10.3389/fbioe.2022.989099.

4. Zhu, S.; Huang, H.; Liu, D.; Wen, S.; Shen, L.; Lin, Q. Augmented cellular uptake and homologous targeting of exosome-based drug loaded IOL for posterior capsular opacification prevention and biosafety improvement. *Bioact Mater* **2022**, *15*, 469-481, doi:10.1016/j.bioactmat.2022.02.019.

5. Huang, X.; Wang, Y.; Cai, J.P.; Ma, X.Y.; Li, Y.; Cheng, J.W.; Wei, R.L. Sustained release of 5-fluorouracil from chitosan nanoparticles surface modified intra ocular lens to prevent posterior capsule opacification: an in vitro and in vivo study. *J. Ocular Pharmacol. Ther.* **2013**, *29*, 208-215, doi:10.1089/jop.2012.0184.

6. Han, Y.M.; Tang, J.M.; Xia, J.Y.; Wang, R.; Qin, C.; Liu, S.H.; Zhao, X.; Chen, H.; Lin, Q.K. Anti-adhesive and antiproliferative synergistic surface modification of intraocular lens for reduced posterior capsular opacification. *Int J Nanomedicine* **2019**, *14*, 9047-9061, doi:10.2147/ijn.s215802.

7. Zhang, X.; Lai, K.; Li, S.; Wang, J.; Li, J.; Wang, W.; Ni, S.; Lu, B.; Grzybowski, A.; Ji, J.; et al. Drug-eluting intraocular lens with sustained bromfenac release for conquering posterior capsular opacification. *Bioact Mater* **2022**, *9*, 343-357, doi:10.1016/j.bioactmat.2021.07.015.

8. Zhang, X.; Wang, J.; Xu, J.; Xu, W.; Zhang, Y.; Luo, C.; Ni, S.; Han, H.; Shentu, X.; Ye, J.; et al. Prophylaxis of posterior capsule opacification through

autophagy activation with indomethacin-eluting intraocular lens. *Bioact Mater* **2023**, *23*, 539-550, doi:10.1016/j.bioactmat.2022.11.024.

9. Lu, D.; Han, Y.; Liu, D.; Chen, S.; Qie, J.; Qu, J.; Lin, Q. Centrifugally concentric ring-patterned drug-loaded polymeric coating as an intraocular lens surface modification for efficient prevention of posterior capsular opacification. *Acta Biomater* **2022**, *138*, 327-341, doi:10.1016/j.actbio.2021.11.018.
10. Ongkasin, K.; Masmoudi, Y.; Wertheimer, C.M.; Hillenmayer, A.; Eibl-Lindner, K.H.; Badens, E. Supercritical fluid technology for the development of innovative ophthalmic medical devices: drug loaded intraocular lenses to mitigate posterior capsule opacification. *Eur J Pharm Biopharm* **2020**, *149*, 248-256, doi:10.1016/j.ejpb.2020.02.011.
11. Xia, J.; Lu, D.; Han, Y.; Wang, J.; Hong, Y.; Zhao, P.; Fang, Q.; Lin, Q. Facile multifunctional IOL surface modification via poly(PEGMA-co-GMA) grafting for posterior capsular opacification inhibition. *RSC Adv* **2021**, *11*, 9840-9848, doi:10.1039/d1ra00201e.
12. Liu, S.; Zhao, X.; Tang, J.; Han, Y.; Lin, Q. Drug-eluting hydrophilic coating modification of intraocular lens via facile dopamine self-polymerization for posterior capsular opacification prevention. *ACS Biomater Sci Eng* **2021**, *7*, 1065-1073, doi:10.1021/acsbiomaterials.0c01705.
13. Topete, A.; Tang, J.; Ding, X.; Filipe, H.P.; Saraiva, J.A.; Serro, A.P.; Lin, Q.; Saramago, B. Dual drug delivery from hydrophobic and hydrophilic intraocular lenses: in-vitro and in-vivo studies. *J Control Release* **2020**, *326*, 245-255, doi:10.1016/j.jconrel.2020.07.020.
14. Lei, M.; Peng, Z.; Dong, Q.; He, Y.; Zhang, Z.; Zhang, X.; Yan, M.; Zhao, C. A novel capsular tension ring as local sustained-release carrier for preventing posterior capsule opacification. *Biomaterials* **2016**, *89*, 148-156, doi:10.1016/j.biomaterials.2016.02.038.
15. Chennamaneni, S.R.; Mamalis, C.; Archer, B.; Oakey, Z.; Ambati, B.K. Development of a novel bioerodible dexamethasone implant for uveitis and postoperative cataract inflammation. *J Control Release* **2013**, *167*, 53-59.

16. Yan, T.Y.; Ma, Z.X.; Liu, J.J.; Yin, N.; Lei, S.Z.; Zhang, X.X.; Li, X.D.; Zhang, Y.; Kong, J. Thermoresponsive genisteinNLC-dexamethasone-moxifloxacin multi drug delivery system in lens capsule bag to prevent complications after cataract surgery. *Sci Rep* **2021**, *11*, 13, doi:10.1038/s41598-020-80476-x.
17. Guha, R.; Chowdhury, S.; Palui, H.; Mishra, A.; Basak, S.; Mandal, T.K.; Hazra, S.; Konar, A. Doxorubicin-loaded MePEG-PCL nanoparticles for prevention of posterior capsular opacification. *Nanomedicine (Lond)* **2013**, *8*, 1415-1428, doi:10.2217/nnm.12.175.
